# 

**Published:** 1873-03

**Authors:** 


### Contents of March Number.

### ORIGINAL DEPARTMENT.

ART. I.—Ten Cases of Excision of Joints. By Alfred T. Livingston, A. B. 281
ART. II.—A Recent Suit for Malpractice. Reviewed by W. F. Hutchinson, M. D. .......... 290
ART. III.—Albany County Medical Society, Semi-Monthly Meeting. Reported by F. C. Curtis, M. D. .......... 300

### MISCELLANEOUS.

Clinical Notes on the Electric Cautery in Uterine Surgery .......... 304

### CORRESPONDENCE.

Oneida County Medical Society .......... 315

### EDITORIAL DEPARTMENT.

Commencement Exercises Buffalo Medical College .......... 316
American Medical Association .......... 317
Obituary .......... 318
Oneida County Medical Society .......... 318
Electrical Instruments .......... 318

BOOKS REVIEWED:

A Manual of Histology. By Prof. S. Stricker .......... 319

Books and Pamphlets Received .......... 320

